# Current Research Advances and Future Prospects on Microbial Consortia for Sustainable PFAS Remediation

**DOI:** 10.3390/ijms27042084

**Published:** 2026-02-23

**Authors:** Hafiz Abdul Kareem, Mohd Faheem Khan

**Affiliations:** 1 Key Laboratory for Space Bioscience and Biotechnology, School of Life Sciences, Northwestern Polytechnical University, Xi’an 710129, China; 2 UCD School of Agriculture and Food Science, University College Dublin, D04 V1W8 Dublin, Ireland

**Keywords:** soil, microbial consortia, PFAS, enzymes, bioremediation, biodegradation

## Abstract

Soil contamination by per- and polyfluoroalkyl substances (PFAS) represents a pressing environmental and public health concern due to the exceptional persistence of carbon–fluorine bonds, which prevent natural attenuation and limit the effectiveness of conventional remediation. Agricultural and industrial soils serve as long-term sinks for PFAS, continuously releasing these pollutants into groundwater and facilitating their transfer through the food chain. Conventional chemical and physical remediation methods are often costly, energy-intensive, and yield incomplete removal, underscoring the need for sustainable and biologically driven alternatives. Microbial consortia have emerged as a promising solution due to their metabolic complementarities, cross-feeding interactions, and ecological resilience, which together enable PFAS transformation and partial defluorination under complex soil and subsurface conditions. Key enzymes such as oxygenases, reductive dehalogenases, and hydrolases are often operating within co-metabolic networks, which play central roles in these processes. Advances in metagenomics, CRISPR-based functional screening, and metabolic modelling are rapidly uncovering novel PFAS-degrading microbes and pathways. Integration of machine learning with multi-omics and environmental datasets further enables the prediction of degradation mechanisms, identification of keystone degraders, and rational design of synthetic consortia. Emerging sustainable strategies, including biochar- and nutrient-amended soil microcosms, plant–microbe partnerships for coupled soil–groundwater phytoremediation, and bioelectrochemical systems that offer new avenues for enhancing PFAS biodegradation in situ. This review synthesises recent research progress and provides critical perspectives on the mechanistic, ecological, and engineering dimensions of PFAS bioremediation, proposing an integrated conceptual framework linking microbial consortia dynamics, enzymatic pathways, and environmental engineering interventions to guide scalable field applications and sustainable management of PFAS-contaminated soil–groundwater ecosystems.

## 1. Introduction

Per- and polyfluoroalkyl substances (PFAS) have emerged as globally pervasive environmental contaminants due to their extensive industrial applications and diffuse entry routes into the environment through sewage sludge, compost, pesticides, and irrigation waters [1]. PFAS contamination now spans water, soil, air, and biota, leading to bioaccumulation across trophic levels. Human exposure is associated with adverse health outcomes, including elevated cholesterol, immune suppression, endocrine disruption, reproductive and developmental toxicity, and increased risks of kidney and testicular cancers [2]. Their extraordinary persistence, stemming from their strong carbon–fluorine (C–F) bonds, has earned them the moniker “forever chemicals,” underscoring their resistance to degradation and long-term accumulation in ecosystems. Point sources such as manufacturing facilities, airports, and military training sites create localised contamination hotspots, while the chain-length-dependent mobility of PFAS allows short-chain analogues to infiltrate groundwater and crops, amplifying risks of food-chain transfer [3]. The updated definition of the organisation for economic co-operation and development (OECD) now classifies PFAS as any molecule containing at least one perfluorinated methyl (−CF_3_) or methylene (−CF_2_−) group [4], reflecting their chemical and toxicological diversity.

Agricultural soils have recently been recognised as both major sinks and secondary sources of PFAS due to long-term inputs from biosolids, wastewater irrigation, and atmospheric deposition [5]. PFAS persist in these soils for decades, and their sorption, transport, and transformation are strongly governed by soil organic matter, mineral composition, texture, and pH, which collectively determine their mobility and bioavailability [6]. Sandy soils, for instance, exhibit higher PFAS leaching than clay-rich systems, due to weaker sorption and greater hydraulic conductivity, ultimately influencing PFAS migration within soil profiles and into underlying compartments. Emerging analytical and modelling approaches, such as LC-MS/MS and HYDRUS simulations, now enable detailed tracking of PFAS fluxes and prediction of long-term accumulation patterns [5]. Among various mitigation strategies, biochar amendment and bioremediation have shown the most promise in limiting PFAS persistence, though regulatory inconsistencies still hinder their large-scale implementation.

**Figure 1:**
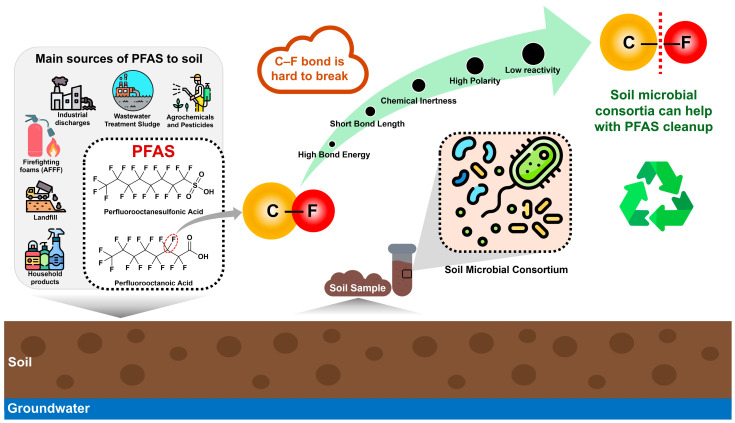
**Main sources and chemical stability of PFAS in soil–groundwater ecosystems**. This figure illustrates the primary sources of PFAS contamination in soils and highlights the role of strong C–F bonds in conferring chemical inertness, high bond energy, short bond length, and low reactivity. Soil microbial consortia are emphasised as a potential strategy to help overcome these challenges in PFAS remediation.

The chemical resilience of PFAS is rooted in the exceptional strength and polarity of the C–F bond, which exhibits a bond dissociation energy of approximately 485 kJ mol^−1^ and a bond length of 1.35 Å. These characteristics render PFAS highly resistant to both abiotic and biotic degradation. Furthermore, the unfavourable thermodynamics of C–F bond cleavage and the hydrophobic–lipophobic nature of fluorinated chains restrict microbial uptake and enzymatic access [7]. Consequently, PFAS demonstrate high recalcitrance and bioaccumulation potential, posing significant challenges for natural attenuation and engineered remediation in soil environments (Figure 1). Beyond chemistry, however, evolutionary constraints in microbial enzymatic repertoires are increasingly recognised as major barriers to efficient biodegradation [8].

Recent advances have deepened our understanding of the microbial and enzymatic mechanisms governing PFAS biotransformation. While individual bacterial isolates often achieve only partial degradation under specific conditions [9], microbial consortia display enhanced transformation capacities through cooperative metabolism. For instance, *Acidimicrobium* sp. A6 has been shown to degrade ~50% of select PFAS derivatives under anaerobic conditions [10,11,12], while mixed communities containing *Paracoccus*, *Hyphomicrobium*, and *Micromonosporaceae* achieved up to 74% removal of perfluorobutanesulfonic acid (PFBS) and 3,3,3-trifluoropropionic acid [13]. Moreover, soil microbiomes enriched on various carbon substrates have demonstrated defluorination of fluorotelomer alcohols and sulfonates via *Variovorax* and *Pseudomonas*-associated pathways [14], and anaerobic perfluorooctanesulfonic acid (PFOS) biodegradation has been linked to *Desulfosporosinus* and *Sulfurospirillum* through desulfonation mechanisms [15]. In wastewater systems, cytochrome P450-mediated oxidation and laccase-catalysed decarboxylation can achieve up to 95% defluorination of perfluorooctanoic acid (PFOA) [16,17]. These findings indicate that PFAS biotransformation is mechanistically diverse, relying on complex interactions between microbial enzymes and environmental conditions.

Consequently, soil ecosystems serve not only as PFAS reservoirs but also as dynamic biogeochemical reactors, where microbial consortia mediate the sorption, transformation, and redistribution of contaminants [5]. Within soil systems, soil–PFAS–microbe interactions are pivotal for developing predictive, sustainable, and field-relevant bioremediation strategies.

Microbial consortia-based bioremediation represents a promising and sustainable approach owing to their inherent functional redundancy, metabolic complementarity, and resilience under variable soil conditions [18]. Through synergistic processes such as cross-feeding of intermediates and community-level adaptation, consortia can outperform pure cultures in degrading recalcitrant contaminants. Most reviews on PFAS remediation focus on physicochemical treatments or microbial degradation in isolation, with limited discussion of enzymatic mechanisms within consortia or integration with computational and engineering tools. This review addresses these gaps by linking microbial community ecology with enzyme-mediated defluorination, incorporating advances in multi-omics, machine learning, and metabolic modelling for PFAS biodegradation prediction, and evaluating engineered bioremediation strategies, including bioelectrochemical systems and plant–microbe partnerships in soil. We also propose a unified framework to guide future research and field-scale implementation of PFAS bioremediation.

## 2. Soil Microbial Consortia and PFAS Degradation Potential

Soil microbial consortia offer a promising approach to tackle persistent PFAS, especially PFOS [19]. The strong C–F bonds and fluorine shielding make PFOS resistant to natural degradation, leading to its widespread presence in soils, water, and biota [7]. Conventional methods like adsorption and advanced oxidation are costly and may produce secondary pollutants. Individual bacterial isolates show limited PFOS degradation due to metabolic constraints and environmental sensitivity [20]. In contrast, microbial consortia, with functional redundancy, complementary pathways, and stress resilience, are well-suited to degrade these persistent pollutants [18].

### 2.1. Natural Microbial Consortia and Adaptation to PFAS Stress in Contaminated Soils

The persistent presence of PFAS in soils exerts strong selective pressure on microbial communities, fostering the development of natural consortia that can tolerate, adapt to, and in some cases, transform these recalcitrant compounds. Unlike individual isolates, diverse native microbial assemblages interact synergistically, overcoming metabolic bottlenecks and enhancing functional stability through complementary metabolic capacities [9,20]. Investigating these communities provides essential insight into naturally evolved adaptation mechanisms and can inform the design of synthetic or enriched consortia for PFAS bioremediation [9].

Metagenomic investigations of PFAS-contaminated soils have revealed substantial but variable shifts in microbial community structure, reflecting the strong yet context-dependent selective pressures imposed by PFAS exposure [21]. Rather than uniformly reducing microbial diversity, PFAS contamination can lead to both enrichment and diversification of certain taxa depending on PFAS type, concentration, and exposure duration [22,23,24]. For example, ether-PFAS exposure was shown to significantly increase species diversity and alter the abundance of nitrogen-cycle-related genes in soil–microbe–plant systems [23]. Similarly, field and batch microcosm studies in PFAS-contaminated agricultural topsoil demonstrated that short-chain perfluoroalkyl acid (PFAA) generation was associated with higher microbial richness and diversity indices compared to uncontaminated controls [24].

A recurrent observation across studies is the enrichment of metabolically versatile and stress-tolerant genera, including *Pseudomonas*, *Rhodococcus*, *Acidimicrobium*, and *Sphingomonas*, which are well known for their capacities to degrade hydrocarbons and halogenated pollutants [10,25]. This enrichment indicates adaptive responses involving enhanced efflux systems, modified membrane composition, and metabolic flexibility that enable survival under PFAS-induced stress and potential co-metabolic transformation of these compounds [8,26].

At the cellular level, long-chain perfluoroalkyl carboxylic and sulfonic acids induce profound molecular and metabolic adaptations. Bacteria exposed to PFAS exhibit altered fatty acid biosynthesis, including increased proportions of medium- to long-chain and monounsaturated fatty acids, which enhance membrane fluidity and stability under chemical stress [27,28]. Recent metabolomic studies further reveal that certain strains, such as *Pseudomonas* sp. 273, can incorporate fluorinated carbon into their lipid bilayers during growth on fluorinated alkanes, resulting in the formation of fluorinated long-chain fatty acids (C_12_–C_19_) and phospholipids, which are up to 82% of total lipids in cells grown with difluorodecane [29]. This incorporation suggests that PFAS or their precursors may partition into and integrate with bacterial membranes, creating intracellular sinks that influence PFAS bioaccumulation, transport, and detoxification.

Extended PFAS exposure also triggers compositional restructuring of microbial communities, characterised by the enrichment of tolerant taxa such as *Bacteroidetes*, *Proteobacteria*, and *Acidobacteria*, alongside declines in more sensitive groups like *Actinobacteria* and *Chloroflexi* [30,31]. This restructuring reflects both ecological selection and functional adaptation, where tolerant populations exploit metabolic plasticity and cell-wide regulatory responses to sustain growth and energy metabolism under PFAS stress. Collectively, these cellular and community-level adjustments highlight the dual processes of physiological acclimation and microbial selection that underpin the persistence and functional resilience of microbial consortia in PFAS-contaminated environments.

### 2.2. Microbial Consortia and Enzymatic Mechanisms in PFAS Bioremediation

**Figure 2:**
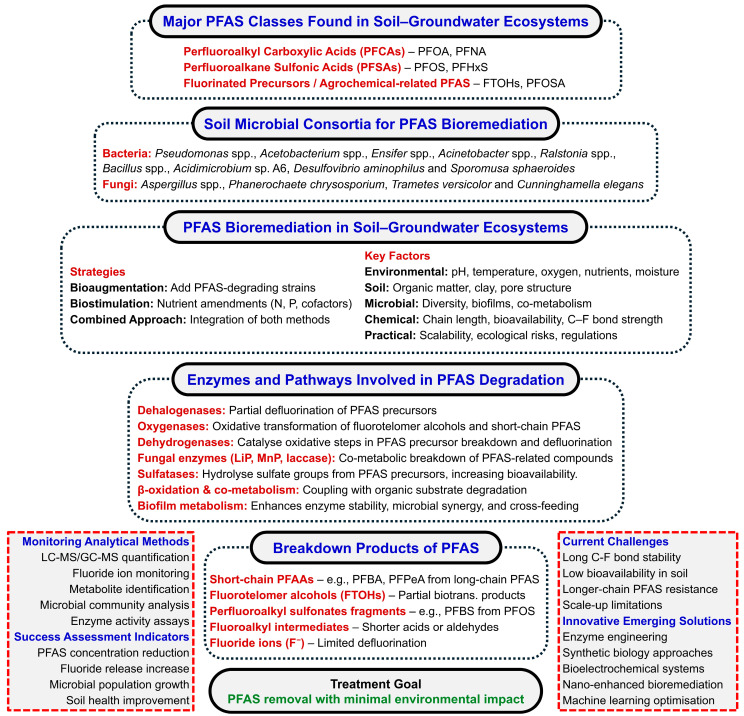
**Overview of PFAS bioremediation in soil–groundwater ecosystems.** The figure shows major PFAS classes and the bacterial and fungal consortia that degrade them. Key strategies, including bioaugmentation, biostimulation, and combined approaches, are illustrated along with environmental, soil, microbial, and chemical factors affecting efficacy. Enzymes and pathways involved in PFAS transformation, resulting products, monitoring methods, and current challenges with emerging solutions are also highlighted.

Microbial consortia play a pivotal role in PFAS bioremediation by facilitating synergistic and complementary metabolic interactions that enable the transformation of otherwise highly recalcitrant compounds, as illustrated in Figure 2. These consortia comprise diverse bacterial genera, including *Pseudomonas*, *Acinetobacter*, *Ralstonia*, and *Desulfovibrio*, in conjunction with fungal taxa such as *Aspergillus*, *Phanerochaete chrysosporium*, and *Trametes versicolor*, which collectively contribute oxidative, reductive, and hydrolytic activities that enhance PFAS biotransformation [32].

Targeted bioaugmentation with specialised PFAS-degrading microorganisms and biostimulation through the amendment of essential nutrients (e.g., nitrogen, phosphorus, and metabolic cofactors) represent widely adopted strategies for improving remediation performance. The integrated application of these approaches has been shown to increase treatment efficiency across heterogeneous environmental and soil matrices [33]. The effectiveness of microbial-mediated PFAS degradation is further modulated by key physicochemical and ecological parameters, including pH, redox conditions, temperature, moisture content, and microbial community composition.

At the molecular level, enzymatic systems such as dehalogenases, oxygenases, and dehydrogenases mediate partial defluorination and oxidative modification of PFAS precursors, thereby initiating transformation processes. In parallel, extracellular fungal enzymes, including lignin peroxidase (LiP), manganese peroxidase (MnP), and laccase, as well as sulfatases, facilitate co-metabolic degradation pathways [34,35]. The resulting transformation products frequently comprise short-chain perfluoroalkyl acids (PFAAs), fluorotelomer alcohols (FTOHs), and inorganic fluoride ions, reflecting incomplete yet progressive defluorination.

Comprehensive assessment of remediation efficacy is supported by advanced analytical methodologies, including liquid chromatography–tandem mass spectrometry (LC–MS/MS), fluoride ion quantification, and high-throughput microbial community profiling. Despite recent advances, significant challenges persist, particularly with respect to the high chemical stability of long-chain PFAS, limited bioavailability, and constraints associated with field-scale implementation [36]. Emerging technological innovations, encompassing enzyme engineering, synthetic biology, bioelectrochemical remediation platforms, and machine learning–assisted process optimization, offer promising avenues for enhancing the efficiency, predictability, and sustainability of PFAS bioremediation with minimal environmental disturbance.

#### 2.2.1. Major PFAS Classes and Occurrence in Soils

PFAS detected in soils encompass a broad spectrum of chemical classes, including perfluoroalkyl carboxylic acids (PFCAs) such as perfluorooctanoic acid (PFOA) and perfluorononanoic acid (PFNA), perfluoroalkyl sulfonic acids (PFSAs) such as perfluorooctane sulfonate (PFOS) and perfluorohexane sulfonate (PFHxS), and numerous fluorinated precursors such as fluorotelomer alcohols (FTOHs) and polyfluorinated agrochemicals used as pesticide adjuvants and active ingredients [8]. Building on the OECD definition introduced above, PFAS are broadly defined as molecules containing at least one perfluorinated methyl (−CF_3_) or methylene (−CF_2_-) group [4], which substantially expands the regulatory scope and complicates risk assessment for agricultural chemicals [37]. This expanded classification underscores the challenge of designing remediation strategies for structurally diverse PFAS compounds.

Anthropogenic inputs such as biosolid application, wastewater irrigation, PFAS-containing agrochemicals, and atmospheric deposition remain the dominant drivers of PFAS accumulation in soils [26,38]. Soil physicochemical properties, including organic matter content, pH, cation exchange capacity, and mineralogy, critically govern PFAS retention and mobility. Importantly, these factors create heterogeneous bioavailability landscapes that can either constrain or enhance microbial access, representing a key but underexplored determinant of bioremediation efficiency. Understanding these soil–PFAS–microbe interactions is therefore essential for designing effective in situ biodegradation strategies.

#### 2.2.2. Soil Microbial Consortia Involved in PFAS Biotransformation

Microbial consortia in soils, comprising both bacteria (e.g., *Pseudomonas* spp., *Acetobacterium* spp., *Ensifer* spp., *Acinetobacter* spp., *Ralstonia* spp., *Acidimicrobium* sp. A6, *Desulfovibrio aminophilus*, *Sporomusa sphaeroides*) and fungi (e.g., *Aspergillus* spp., *Phanerochaete chrysosporium*, *Trametes versicolor*, *Cunninghamella elegans*), have demonstrated significant potential for PFAS biotransformation. These consortia offer functional redundancy, metabolic versatility, and ecological resilience, attributes often absent in single isolates [20]. Bioremediation strategies include bioaugmentation, biostimulation through nutrient amendments (e.g., nitrogen, phosphorus, cofactors), and combined approaches. The success of these strategies largely depends on the characteristics and interactions of the microbial communities themselves, including diversity, consortia stability, biofilm formation, and co-metabolic capabilities.

#### 2.2.3. Factors Influencing PFAS Biodegradation

Environmental, soil, microbial, chemical, and practical factors collectively govern PFAS biodegradation in soils. Redox conditions, pH, temperature, and moisture strongly influence microbial activity and PFAS bioavailability, with aerobic and anaerobic pathways differing in efficiency and optimal degradation often occurring at pH 6–7 and 25–35 °C [32]. Soil characteristics such as organic matter content, texture, and ageing affect PFAS adsorption and mobility, where high organic carbon can sequester PFAS, reducing bioavailability, and clay-rich soils provide more sorption surfaces [5,24]. Microbial factors, including diversity, consortia formation, adaptation, and enzyme activity (e.g., dehalogenases), determine transformation rates, with tolerant taxa such as *Pseudomonas* and *Acidimicrobium* sp. playing key roles under selective pressure [25,26]. Chemical properties, including PFAS chain length, headgroup chemistry, precursors, co-substrates, and co-contaminants, influence enzymatic accessibility, solubility, and competitive interactions that affect degradation [38,39]. Practical and engineering factors, such as bioaugmentation, bioreactors, hybrid systems, and amendments (e.g., biochar, iron-based materials), can enhance microbial activity and PFAS sequestration; however, adsorption effects must be carefully distinguished from true biodegradation to avoid misinterpretation [39]. Together, these factors highlight the need for site-specific optimisation and integrated assessment to achieve reliable PFAS bioremediation outcomes.

#### 2.2.4. Enzymatic Mechanisms of PFAS Biodegradation

**Figure 3:**
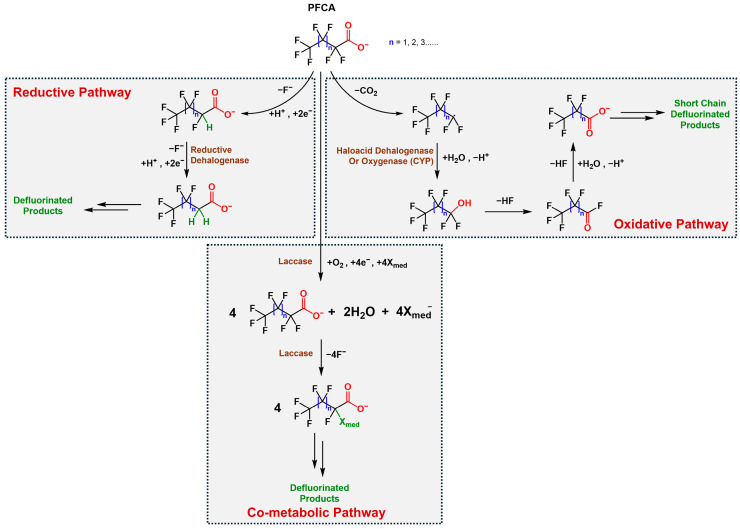
**Schematic representation of PFAS (PFCA) biodegradation pathways showing oxidative, reductive, and co-metabolic mechanisms.** Oxidative processes (right) involve hydroxylation and subsequent C–F cleavage catalysed by haloacid dehalogenases or oxygenases (e.g., CYPs), generating short-chain defluorinated intermediates. Reductive mechanisms (left) are mediated by reductive dehalogenases, which sequentially replace fluorine atoms with hydrogen under electron-donating conditions. Co-metabolic routes (bottom) employ fungal laccases that utilise external mediators (X_med_) and oxygen to catalyse partial defluorination, producing smaller carboxylates and releasing fluoride ions.

The biodegradation of PFAS compounds involves diverse enzymatic and co-metabolic mechanisms, though the extreme stability of the carbon–fluorine (C–F) bond presents significant challenges. Defluorinase enzymes, including hydrolases, oxidoreductases, and dehalogenases, have been identified, offering multiple routes for aerobic, anaerobic, and co-metabolic transformation of PFAS precursors and derivatives [40]. Figure 3 illustrates three principal mechanisms: oxidative, reductive, and co-metabolic pathways involved in PFAS degradation.

- **Oxygenase- and Haloacid Dehalogenase-Mediated (Oxidative) Pathways**

Oxygenase and haloacid dehalogenase (HAD)-mediated reactions constitute central oxidative and hydrolytic routes for PFAS transformation. Oxygenases, including monooxygenases, cytochrome P450s, and peroxygenases, introduce oxygen into PFAS precursors, initiating chain-shortening and partial defluorination [41,42,43]. Microbial consortia containing *Pseudomonas* and *Rhodococcus* spp. oxidise fluorotelomer alcohols (FTOHs) to fluorotelomer aldehydes, fluorotelomer carboxylic acids (FTCAs), and ultimately perfluoroalkyl acids (PFAAs) such as PFOA. These transformations are key aerobic processes linking PFAS precursors to more persistent terminal products [44].

Haloacid dehalogenases, a subclass of hydrolases, catalyse C–F bond cleavage in haloalkanoic acids through a two-step SN2 substitution mechanism involving an enzyme–ester intermediate and nucleophilic water attack. Fluoroacetate dehalogenases, identified in *Pseudomonas*, *Burkholderia*, *Delftia*, and *Rhodopseudomonas* spp., utilise conserved catalytic triads (Asp–His–Asp) and hydrogen-bond networks formed by residues such as Trp150 and Tyr212 to stabilise transition states, enabling efficient fluoride release [26]. These enzymes exhibit remarkable substrate flexibility, defluorinating compounds like 2-fluoropropionic acid, difluoroacetate, and 6:2 fluorotelomer precursors (POL0530, RJO0230; RPA1163) [45,46]. Engineered *E. coli* strains expressing HAD genes have demonstrated limited PFOA defluorination, supporting their potential for bioremediation [9,47].

Together, oxygenase-driven oxidation and HAD-mediated hydrolysis constitute complementary mechanisms that initiate C–F bond cleavage, generate short-chain defluorinated intermediates, and promote aerobic microbial adaptation to PFAS-contaminated environments.

- **Reductive Dehalogenase-Mediated (Anaerobic) Mechanisms**

Reductive dehalogenases (RDases) catalyse electron-driven defluorination under anaerobic conditions, forming a key mechanism for PFAS biodegradation in oxygen-limited environments. These enzymes are broadly categorised as respiratory RDases, membrane-bound and oxygen-sensitive, or catabolic RDases, which are cytoplasmic and oxygen-tolerant [48,49]. Both classes contain corrinoid (cobalamin) and [4Fe–4S] clusters that mediate electron transfer via cobalt–halide coordination, enabling substrate reduction [26,48,50].

Although enzymatic reductive defluorination remains challenging due to the high bond dissociation energy of the C–F bond (ΔG° > 500 kJ mol^−1^), evidence is emerging. *Cloacibacillus porcorum* MFA1 stoichiometrically defluorinates monofluoroacetate (MFA) to acetate, while *Acidimicrobium* sp. A6 degrades PFOA and PFOS using hydrogen or ammonium as electron donors and Fe(III) as an acceptor [48]. Metagenomic analyses revealed a partial reductive dehalogenase gene (A6RdhA) in strain A6, with homologues (T7RdhA) identified in marine metagenomes encoding Fe–S–S proteins with norpseudo-B_12_ cofactors [7,26,51].

*Dehalococcoides*-containing consortia (KB1) have shown vitamin B_12_-dependent defluorination of unsaturated PFAS, albeit without confirmed RDase gene transcription [49]. Hybrid systems integrating bioelectrochemical processes with defluorinating microbes (*Desulfovibrio*, *Sporomusa*) enhanced C–F bond cleavage, underscoring the synergistic potential of redox-active materials and microbial catalysis [7,26,52].

Collectively, reductive dehalogenases represent promising enzymatic tools for PFAS remediation. Advancing structural, electrochemical, and synthetic biology research could enable harnessing these enzymes for targeted, energy-efficient defluorination in anoxic soil and groundwater environments.

- **Other Enzymes and Co-metabolic Processes**

Beyond oxygenases and reductive dehalogenases, several auxiliary enzymes contribute indirectly to PFAS transformation through co-metabolic processes. Dehydrogenases catalyse oxidative reactions involved in PFAS precursor degradation, particularly transforming fluorotelomer alcohols and carboxylates into shorter-chain metabolites. Fungal oxidative enzymes, including laccases, lignin peroxidase (LiP), and manganese peroxidase (MnP), have also been implicated in non-specific PFAS transformation, often through radical-mediated oxidation of C–F-containing substrates [51]. These enzymes typically act fortuitously during the metabolism of co-substrates such as glucose, methane, or propane, a process that supports incidental defluorination [53].

Recent studies indicate that fungal laccases (e.g., from *Trametes versicolor*) possess redox potentials (≈470–800 mV vs. Normal Hydrogen Electrode) that are insufficient for direct PFAS oxidation; however, laccase–mediator systems can generate reactive radicals capable of transforming PFAS via indirect oxidation pathways. Experimental studies have reported partial PFOS and PFOA degradation, although sorption effects have also been observed, and enzymatic defluorination remains limited [54].

Co-metabolic processes involving ammonium oxidation have been shown to stimulate defluorination by *Acidimicrobium* sp. A6, suggesting a mechanistic coupling between nitrogen cycling and PFAS turnover [48]. Such findings highlight the potential of using targeted biostimulation strategies, through co-substrate or nutrient amendment, to enhance PFAS biodegradation in situ by promoting redox cycling and enzyme activation within soil microenvironments.

Furthermore, β-oxidation and desaturation pathways may integrate PFAS-derived intermediates into broader lipid metabolism, sustaining microbial activity under nutrient limitation or contaminant stress [55]. Understanding these auxiliary enzymatic and metabolic linkages offers promising avenues for developing multi-enzyme, community-based PFAS bioremediation strategies.

#### 2.2.5. Transformation Pathways and Monitoring of PFAS Biodegradation

PFAS transformation produces a series of intermediates, short-chain PFAAs, FTOHs, perfluoroalkyl sulfonate fragments (e.g., PFBS from PFOS), fluoroalkyl aldehydes, and fluoride ions [36,50]. Partial degradation can yield shorter-chain PFAS and intermediate fluorinated compounds that may exhibit enhanced toxicity, bioaccumulation, and endocrine-disrupting effects, as demonstrated for PFBA, PFPeA, PFBS, PFHxA, PFHxS, and related compounds in aquatic and terrestrial organisms [56]. Trifluoroacetate (TFA) is of particular concern as it is an environmentally persistent and mobile end-product arising from both abiotic oxidation and microbial transformation [42]. TFA accumulation represents a key indicator of incomplete PFAS degradation and legacy pollution in soils and waters, warranting its inclusion in all PFAS fate assessments.

Short-chain PFAS transformation products are generally more soluble and less sorptive to soils and sediments, which increases their groundwater mobility and potential for trophic transfer across ecosystems. Some volatile intermediates, such as FTOHs, may also contribute to atmospheric transport and long-range redistribution. These changes in physicochemical properties can increase environmental exposure risks despite reductions in parent PFAS concentrations [56].

Analytical approaches for monitoring PFAS biotransformation include LC–MS/MS and GC–MS quantification, fluoride ion monitoring, metabolite identification, microbial community profiling, and enzyme activity assays [57,58]. Success indicators encompass PFAS concentration reduction, fluoride release, microbial enrichment, and soil health recovery. However, monitoring should also include toxicity-based endpoints and bioaccumulation assessments to evaluate unintended risk associated with transformation products.

Despite notable progress, long-chain PFAS remain highly resistant to biological breakdown due to their chemical stability and low soil bioavailability. The scale-up of bioremediation systems is further constrained by ecological and regulatory complexities [44]. However, innovative approaches, including enzyme engineering, synthetic biology, bioelectrochemical systems, nano-enabled remediation, and machine learning-guided optimisation, offer new opportunities to enhance PFAS degradation kinetics and selectivity across diverse soil environments [59].

Table 1 summarises reported studies on the biodegradation of PFAS by microbial consortia and individual strains, highlighting their sources, experimental conditions, degradation products, and efficiencies.

### 2.3. Synergistic Interactions in Consortia

The effectiveness of a microbial consortium arises from emergent properties generated through interactions among its members, rather than the sum of individual capabilities. Such synergies facilitate PFAS degradation by supporting complementary metabolic processes such as co-metabolism, metabolic cross-feeding, and cooperative enzyme expression, thereby broadening the range of substrates that can be transformed within the community [32]. Consortia do not “enable” co-metabolism per se but provide the ecological and biochemical context in which co-metabolic reactions become more efficient through substrate sharing, redox balancing, and mutualistic interactions. Functional redundancy is a characteristic feature of soil microbiomes, which further enhances system robustness, as multiple taxa often encode enzymes with overlapping functions, enabling partial or complete PFAS biotransformation even if specific members are lost [77]. This redundancy is advantageous because PFAS degradation likely involves promiscuous oxygenases, dehalogenases, and reductases capable of acting on diverse fluorinated intermediates, implying that several microbial lineages in soils may collectively catalyse analogous defluorination steps, although such capacity may vary across soil types and contamination histories.

#### 2.3.1. Cross-Feeding of Intermediates

The cross-feeding of metabolic intermediates establishes a cooperative degradation network in which incomplete transformation products generated by one microbial species serve as substrates for others [78]. Such nutrient exchange is a ubiquitous feature of microbial communities, underpinning emergent properties that influence human health and regulate global biogeochemical cycles [79]. In the context of PFAS degradation, cross-feeding is particularly important due to the sequential nature of defluorination reactions [77]. For instance, although Feammox activity in pure and enriched *Acidimicrobium* sp. A6 cultures were comparable in terms of ammonium removal, Fe (II) production, and A6 cell counts; the enrichment culture exhibited enhanced PFOA and PFOS removal, with increased formation of shorter perfluorinated compounds, acetate, and fluoride ions [80]. While this remains one of the few experimental demonstrations, similar consortium-mediated improvements in PFAS degradation efficiency have been reported under mixed-culture and bioaugmentation settings, reinforcing that cross-feeding is an emergent yet ecologically plausible mechanism rather than an isolated observation [32]. This suggests synergistic interactions between A6 and co-occurring organisms, where heterotrophic partners further metabolise partially defluorinated intermediates, thereby enhancing overall PFAS degradation efficiency [81].

#### 2.3.2. Biofilm-Mediated Protection and Stability in Soil Environments

Biofilm formation is a key survival strategy for microbial consortia exposed to PFAS-contaminated soils, providing both physical protection and metabolic advantages. Within biofilms, cross-feeding and diffusional gradients create local metabolic niches that allow slower-growing species to coexist with faster-growing species, stabilising the community against environmental perturbations. Biofilm-associated extracellular polymeric substances (EPS) play multifunctional roles in PFAS bioremediation, including facilitating biosorption rather than direct desulfonation or defluorination, as PFOS removal in Bhagwat et al. resulted primarily from enhanced adsorption onto biofilm-coated plastic fibres rather than enzymatic cleavage [82]. Transcriptomic analyses of biofilm consortia reveal high expression of genes encoding enzymes such as alkanesulfonate monooxygenase, (S)-2-haloacid dehalogenase, and putative cytochrome P450, which support both biofilm formation and PFAS transformation [7,37,83]. However, the constitutive expression of these enzymes within biofilms is unlikely; rather, their induction may occur in response to organic co-substrates or co-contaminants structurally analogous to PFAS precursors. Additionally, catechol dioxygenase primarily acts on aromatic intermediates and is not directly implicated in PFAS defluorination, suggesting that the referenced study likely investigated fluorinated aromatic substrates rather than fully perfluorinated compounds.

Recent evidence further indicates that biofilm-mediated resilience in contaminated soils is strongly regulated by quorum sensing (QS), which coordinates microbial communication, catabolic gene expression, and the spatial organisation of degradative enzymes [84]. By synchronising enzyme production and enhancing cooperative metabolism, QS-regulated networks reinforce biofilm stability and can substantially increase the efficiency of pollutant degradation. Moreover, incorporating QS-mediated interactions into the design of microbial consortia is emerging as a promising strategy to enhance biofilm robustness and optimise PFAS biotransformation in complex soil environments. The protective and structural functions of biofilms are further enhanced by porous matrices such as biochar and activated carbon, which adsorb PFAS while providing surfaces for microbial colonisation. These bioactive zones, particularly around plant roots, promote consortia stability and maintain high biotransformation activity in soil environments [85].

### 2.4. Case Studies on PFAS Occurrence and Microbial Biodegradation in the Soil Environment

Several case studies highlight the persistence and environmental relevance of PFAS contamination in soils and water-associated matrices, as well as the variable potential for microbial bioremediation. Kim and co-workers reported that a soil-derived microbial consortium comprising *Mycobacterium vaccae* JOB5, *Pseudomonas oleovorans*, *Pseudomonas butanovora*, and *Pseudomonas fluorescens* DSM 8341 achieved up to 88% degradation of 6:2 fluorotelomer alcohol (6:2 FTOH), producing shorter-chain perfluoroalkyl acids such as PFHxA and PFBA [61]. *Acidimicrobium* sp. A6, isolated from acidic wetland soils, was shown by Huang and Jaffé and later by Ruiz-Urigüen et al. to degrade 50–77% of PFOA under anaerobic and electrochemical enrichment conditions, with fluoride release confirming defluorination [10,11].

In another study, *Pseudomonas parafulva* YAB1 isolated from soil near a fluorochemical production facility achieved 48% PFOA degradation under aerobic conditions [63]. Yang et al. demonstrated that *Rhodococcus jostii* RHA1, isolated from contaminated soil, achieved up to 99% transformation of 6:2 FTSA via desulfonation and defluorination pathways [66]. In contrast, long-term anaerobic incubation experiments conducted by Ochoa-Herrera et al. revealed no detectable microbial degradation of PFOS and related PFAS, highlighting the extreme recalcitrance of certain PFAS classes [70]. Shaw et al. reported that *Gordonia* sp. strain NB4-1Y isolated from soil achieved near-complete degradation of 6:2 FTSA and substantial transformation of 6:2 FTAB under sulfur-limited conditions [72].

Collectively, these studies demonstrate both the environmental prevalence of PFAS and the compound-specific variability in microbial degradation potential, underscoring the need for tailored and system-specific bioremediation strategies.

## 3. Recent Advances in PFAS Biodegradation Within Soil Ecosystems

Per- and polyfluoroalkyl substances (PFAS) are among the most recalcitrant environmental contaminants in terrestrial ecosystems because of their extreme chemical stability and resistance to enzymatic attack. Over the past decade, increasing attention has been directed toward understanding microbial processes capable of initiating or mediating PFAS defluorination in soils. Recent research has demonstrated that although complete mineralisation is rare, partial transformations, particularly chain-shortening, desulfonation, and reductive defluorination, can occur under specific environmental and metabolic conditions [86].

This section summarises recent advances in microbial PFAS degradation in soils, with particular emphasis on enrichment and selection approaches, identification of key microbial taxa, and community-level interactions that collectively underpin observed biodegradation activity.

### 3.1. Enrichment and Selection Approaches

Enrichment and selection approaches represent foundational strategies for developing PFAS-degrading microbial communities. These approaches aim to selectively cultivate microorganisms that can tolerate PFAS toxicity while possessing the enzymatic potential for partial or complete defluorination. By applying controlled selection pressures such as exposure to PFAS substrates, co-metabolic amendments, or sequential incubation regimes, microbial populations can be progressively adapted to enhance their degradation capacity [87].

**Figure 4:**
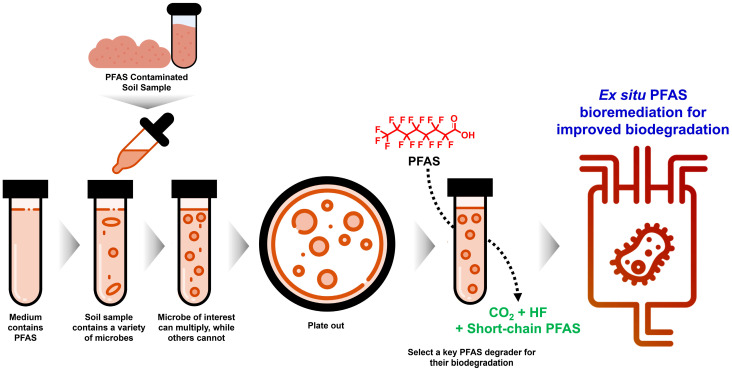
**Enrichment and selection of efficient PFAS degraders.** PFAS-contaminated soils are incubated in selective media to promote the growth of microorganisms capable of metabolising recalcitrant PFAS compounds. Iterative enrichment allows isolation and screening of strains with superior ability to convert PFAS into CO_2_, HF, and short-chain PFAS, supporting ex situ bioremediation.

Such strategies not only improve degradation efficiency but also facilitate the recovery of novel PFAS-transforming taxa and enzymatic pathways from complex environmental inocula. Figure 4 illustrates the workflow for enrichment and selection of native microbial consortia or isolates for PFAS degradation, highlighting the progressive selection of microorganisms with superior defluorination activity.

#### 3.1.1. Sequential Enrichment and Selection of PFAS-Degrading Consortia

Recent enrichment-based studies have provided direct evidence for the existence of microbial communities with measurable PFAS-degrading capacity. Continuous and sequential enrichment approaches, often coupled with the addition of suitable co-metabolic substrates or electron donors, have proven effective in developing microbial consortia with enhanced PFAS-degrading capacities [10,87].

For example, incubations with pure and enrichment cultures of *Acidimicrobium* sp. strain A6, an autotroph that oxidises ammonium while reducing ferric iron, revealed that enrichment cultures consistently outperformed pure cultures, achieving up to 60% removal of PFOA and PFOS within 100 days [39,88]. Similarly, iterative enrichment from environmental inocula under selective pressure has yielded strains such as *Pseudomonas aeruginosa* HJ4, *Ensifer adhaerens* M1, and *Pseudomonas plecoglossicida* 2.4-D, each demonstrating significant PFOS transformation capacity [62].

Sequential enrichment further refines these communities by gradually acclimating them to structurally diverse PFAS substrates. Studies employing mixed PFAS (C4–C10 PFCAs and PFSAs, including branched and linear isomers) showed that *Acidimicrobium* sp. A6 achieved 11.5–56.9% removal over 120 days, with PFOA degradation efficiencies reaching 50% in enrichment cultures compared to 33% in pure cultures [10,89]. These studies collectively underscore that stepwise adaptation enhances substrate versatility, stabilises microbial networks, and improves PFAS biotransformation under soil-relevant conditions.

#### 3.1.2. Optimisation of Co-Metabolic Substrates for Enhanced Defluorination

The availability and type of co-metabolic substrates exert a pivotal influence on PFAS biodegradation during enrichment. Control experiments demonstrated that decreases in PFOS-K levels within the WH4 consortium were primarily attributable to microbial metabolism rather than abiotic effects of methanol addition [18]. However, the presence of easily metabolised non-fluorinated carbon sources may deter PFAS utilisation, as microbes preferentially exploit more labile substrates [90].

The innovation in co-metabolic optimisation lies not in the use of generic carbon supplements but in identifying specific co-substrates or electron donors that induce the expression of oxygenases, reductive dehalogenases, and other PFAS-transforming enzymes. This requires a delicate balance between energy metabolism and enzyme induction; excessive co-substrate availability can suppress PFAS activation or divert electron flow away from defluorination. Thus, precise optimisation of substrate type and concentration is critical to sustain microbial metabolism while directing enzymatic activity toward defluorination. Such tailored co-metabolic regimes are emerging as key levers to improve enrichment outcomes and to promote the transformation of otherwise intractable PFAS molecules.

### 3.2. Identifying Keystone Microbial Degraders Within Soil Consortia

The discovery of microbial taxa capable of defluorination has marked a turning point in PFAS biodegradation research. Identifying keystone species, microorganisms that exert disproportionately important roles in PFAS degradation, is essential for understanding and optimising bioremediation strategies (Figure 4). Advanced molecular and omics-based analyses have revealed several critical degraders within PFAS-exposed soil communities, providing insight into their functional roles and potential for biotechnological applications.

#### 3.2.1. *Acidimicrobium* sp. Strain A6: A Model Keystone Degrader

*Acidimicrobium* sp. strain A6, a recently characterised autotrophic bacterium, oxidises ammonium while reducing ferric iron and is commonly found in acidic, iron-rich soils [91]. Its genome encodes multiple reductive dehalogenases, equipping the strain with enzymatic machinery capable of defluorination. Functional studies demonstrate that *Acidimicrobium* sp. strain A6 actively participates in PFAS degradation: incubations with perfluorooctanoic acid (PFOA) or perfluorooctane sulfonate (PFOS) lead to the release of fluoride ions and shorter chain PFAAs, with concurrent expression of the *rdhA* gene [48].

Targeted gene knockouts have confirmed that *rdhA*-associated enzymes are indispensable for PFAS defluorination [26]. Kinetic analyses further demonstrated a direct correlation between ammonium oxidation rates and defluorination efficiency, indicating a coupled nitrogen–fluorocarbon metabolism. This metabolic interconnection suggests that nutrient management strategies could be exploited to stimulate PFAS degradation [92].

#### 3.2.2. *Pseudomonas* Species as Versatile Degraders

Pseudomonas species represent one of the most metabolically adaptable bacterial genera involved in PFAS degradation, capable of utilising diverse oxidative and hydrolytic pathways. Several strains, including *Pseudomonas aeruginosa*, *Pseudomonas putida*, and *Pseudomonas parafulva*, have demonstrated measurable PFOS and PFOA transformation, often within enrichment consortia containing other soil and rhizosphere bacteria such as *Alternaria*, *Bacillus cereus*, and *Serratia marcescens* [93,94].

Recent evidence highlights *Pseudomonas mosselii* strain 5(3) as a potent degrader of medium-chain perfluorocarboxylic acids (C7–C10 PFCAs) [95]. Genomic analysis revealed key defluorination-related genes, including *dhaA* (haloalkane dehalogenase), *dehH1* (haloacetate dehalogenase H-1), *crcB* (fluoride ion transporter), and *ssuE* (alkanesulfonate monooxygenase). This genetic repertoire supports complete PFCA mineralisation, confirmed by stoichiometric fluoride release.

At the enzymatic level, fluoroacetate dehalogenases (FADs) from *Pseudomonas* and related genera catalyse hydrolytic cleavage of the strong C–F bond under mild conditions [96]. Structural and mechanistic studies of FADs, including DAR3835 and NOS0089, reveal conserved catalytic triads and substrate tunnels enabling the stepwise defluorination of difluoroacetate into glyoxylate, underscoring their substrate promiscuity and biotechnological potential.

Together, these findings position *Pseudomonas* spp. as pivotal taxa in PFAS biodegradation networks, combining genomic plasticity, enzymatic versatility, and environmental adaptability for targeted bioremediation applications.

#### 3.2.3. Keystone Interactions and Functional Guilds in PFAS-Enriched Communities

Keystone functionality within PFAS-degrading consortia often arises from interspecies cooperation rather than individual taxa alone. Microbial community analyses of PFAS-enriched soils have revealed the co-occurrence of denitrifiers (e.g., *Ralstonia*, *Bacillus*), iron reducers (e.g., *Acidimicrobium*, *Aciditerrimonas*), and sulphate reducers (e.g., *Desulfosporosinus*) [97]. These functional guilds form synergistic redox networks, where denitrifiers generate electron acceptors, stimulating iron and sulphate reducers, while the latter provide reducing equivalents that facilitate PFAS defluorination.

PFAS exposure imposes strong selective pressures, leading to community compositional shifts that favour metabolically cooperative guilds. For example, the relative abundance of *Acidimicrobium*, *Paraburkholderia*, and *Desulfosporosinus* increases in cultures enriched with PFCA, PFSA, or PFOA/PFOS, illustrating dynamic keystone relationships that collectively enhance PFAS transformation under stress [98].

In summary, recent investigations provide unequivocal evidence for microbially mediated PFAS transformation in soils, driven by adaptive enrichment, co-metabolic optimisation, and keystone microbial cooperation. Although complete defluorination remains limited, these findings collectively establish that soil microbiomes harbour both the enzymatic potential and ecological diversity necessary for partial PFAS biodegradation. This growing body of evidence forms the foundation for engineered enhancement strategies, which are discussed in the following section.

## 4. Strategies to Enhance PFAS Biodegradation in Soils

Although natural PFAS biodegradation in soils has been demonstrated, the intrinsic metabolic potential of native microbiomes remains insufficient for effective remediation. Consequently, multiple strategies have been developed to accelerate and optimise these processes. These include the enrichment and selection of naturally occurring PFAS-degrading populations, optimisation of co-metabolic processes, and the application of bioaugmentation with specialised strains. More recently, synthetic biology, metabolic engineering, and systems-level optimisation have been proposed to enhance enzymatic activity, substrate specificity, and community resilience [10,87].

While soil microbiomes display high functional redundancy for common carbon and nitrogen cycling pathways, this trait does not directly translate to PFAS degradation, as only a few taxa possess the rare catalytic capabilities necessary for defluorination. Therefore, functional redundancy in the context of PFAS biodegradation is limited and cannot be considered a favourable or defining characteristic of soil microbial systems, necessitating targeted enrichment or bioaugmentation to achieve measurable transformation. Collectively, these approaches aim to transform native microbial consortia into more efficient and adaptable systems for PFAS remediation in complex soil environments.

### 4.1. Engineering and Systems Biology Tools

Beyond natural enrichment, synthetic biology and systems-level modelling offer precision strategies for the rational design of microbial consortia with enhanced PFAS-degrading potential. Synthetic biology can accelerate and optimise PFAS biodegradation by overcoming the slow and unpredictable nature of natural evolution. A wide array of defluorination-associated enzymes, spanning multiple Enzyme Commission classes, constitutes a versatile toolkit for metabolic pathway engineering, enabling the construction of customised microbial platforms for targeted PFAS detoxification [7,99].

**Figure 5:**
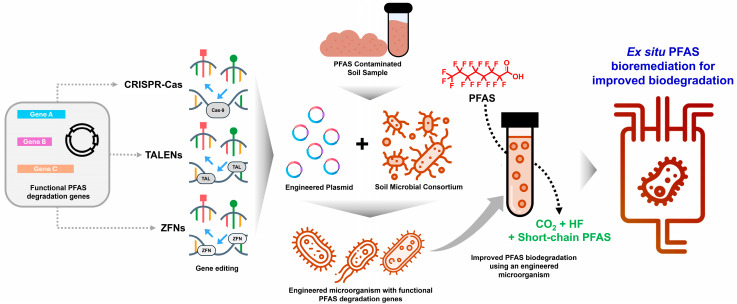
**Engineering and systems biology tools to improve PFAS degradation.** Functional PFAS-degrading genes are introduced into microbial genomes or plasmids using CRISPR-Cas, TALENs, or ZFNs. Engineered strains are combined with native soil consortia and exposed to PFAS substrates, enhancing defluorination and conversion to CO_2_ and HF. This illustrates the potential of gene editing and systems biology to optimise PFAS biodegradation.

As illustrated in Figure 5, engineered microorganisms developed through CRISPR-Cas, TALENs, or ZFN-mediated genome editing can be integrated with native soil consortia to enhance metabolic synergy and ecological resilience. Systems biology tools, including genome-scale metabolic modelling and multi-omics integration, help identify key degraders, predict interactions, and optimise engineered pathways. Together, these strategies enable the translation of laboratory designs into ex situ bioreactors, improving PFAS biodegradation efficiency, stability, and scalability. The focus on ex situ bioremediation reflects the need to avoid biosafety risks linked to the dissemination of antibiotic resistance genes and genetically modified organisms in open environments [100]. In contained bioreactors or treatment units, microbial consortia can be precisely monitored, with biosafety supported by field-adapted consortia, encapsulated formulations, protective carriers, and containment measures. The engineered strains, combined with native soil consortia, are applied to PFAS-contaminated substrates under controlled ex situ conditions, promoting efficient degradation while minimising environmental release of modified organisms and resistance determinants.

#### 4.1.1. CRISPR-Based Genome Engineering

CRISPR/Cas-based genome engineering offers a conceptually powerful but still largely theoretical platform for enhancing microbial PFAS degradation. While no published studies have yet demonstrated direct CRISPR-mediated modification of PFAS-degrading genes or microorganisms, this technology is increasingly recognised for its potential in environmental biotechnology and microbial optimisation [101,102].

Recent reviews emphasise that CRISPR and related genetic engineering tools could accelerate the creation of genetically engineered microorganisms (GEMs) by enabling targeted modification of enzymatic and metabolic pathways involved in fluorinated compound transformation [8,77]. Instead of demonstrated applications, current studies focus on proof-of-concept frameworks, such as transferring or enhancing genes encoding reductive dehalogenases, oxygenases, or hydrolases that show partial defluorination activity [103,104].

CRISPR systems could be employed to upregulate or optimise enzyme expression, or to eliminate competing metabolic pathways, improving substrate utilisation and reducing metabolic burden [61,105]. Moreover, genome engineering holds promise for constructing fluoride-tolerant microbial strains, mitigating the inhibitory effects of fluoride ions released during defluorination and ensuring metabolic stability and sustained catalytic activity.

Olawade et al. highlighted that GEMs, when appropriately contained, offer significant process safety advantages over thermal or chemical PFAS destruction methods. They operate under ambient conditions, reducing risks of explosion or toxic gas formation, and allow controlled biodegradation within closed bioreactors or soil microcosms [102,106]. Nonetheless, deployment faces regulatory, ecological, and scalability challenges, including biocontainment, environmental survivability, and gene transfer monitoring [107]. Although CRISPR-based genome engineering for PFAS degradation remains in its infancy, it represents a promising enabling tool for developing synthetic or hybrid microbial systems capable of safe, efficient, and sustainable PFAS bioremediation under environmentally relevant conditions.

#### 4.1.2. Synthetic Microbial Consortia Design

The rational design of synthetic microbial consortia (SMC) represents a promising avenue for advancing PFAS biodegradation. Such strategies aim to integrate complementary metabolic pathways, enhance community stability, and facilitate efficient defluorination through coordinated microbial interactions. This concept aligns with broader efforts to harness interspecies cooperation for the degradation of persistent organic pollutants. Recent reviews highlight the significant potential of microbial consortia to improve PFAS transformation, while noting that applications involving synthetic or engineered communities have yet to be realised [32].

To date, no study has reported the successful rational design of SMC specifically for PFAS biodegradation or bioremediation. However, the approach remains conceptually valuable and aligns with broader efforts to harness microbial community interactions for the degradation of persistent pollutants. Recent reviews have emphasised the promise of microbial consortia in enhancing PFAS breakdown, while acknowledging the current lack of synthetic or engineered community applications [32].

Existing research has primarily relied on enrichment-based or “top-down” community selection methods rather than rational “bottom-up” design. For instance, Liang and Ma identified a PFOS-degrading microbial consortium through functional screening and enrichment, achieving a 56.7% reduction in PFOS concentration under optimal conditions with *Hyphomicrobium* spp. as key members [18]. Such naturally assembled consortia illustrate that synergistic microbial interactions can promote PFAS transformation, even though the underlying mechanisms remain poorly defined.

In contrast, synthetic consortia design represents a forward-looking strategy, drawing on principles from systems biology and synthetic ecology. The approach involves combining two or more microbial species, either genetically modified or naturally isolated, to perform distributed metabolic tasks collaboratively [10]. Although such designs have yet to be implemented for PFAS, conceptual frameworks for their development have been proposed through data-driven synthetic biology workflows [108]. These involve integrating omics, machine learning, and computational modelling to identify degradation-relevant pathways, engineer enzymes or strains, and assemble synthetic microbial communities capable of cooperative pollutant transformation.

Future applications of SMC in PFAS bioremediation will require bridging current knowledge gaps, including the identification of degradative genes, metabolic intermediates, and stable microbial partnerships that can withstand PFAS toxicity and co-contaminant stress. Integrating experimental enrichment data with computationally guided community design could enable the next generation of PFAS-degrading consortia, transitioning from empirical enrichment to predictive, data-driven engineering. Overall, while no synthetic PFAS-degrading consortia have yet been demonstrated, the convergence of enrichment studies, systems biology, and data-driven synthetic design offers a conceptual roadmap for developing high-performance, adaptive microbial communities for sustainable PFAS remediation.

#### 4.1.3. Genome-Scale Metabolic Modelling

To date, genome-scale metabolic modelling (GSMM) has not been successfully implemented for PFAS biodegradation or bioremediation. Nevertheless, the conceptual framework offers valuable insight for future applications aimed at elucidating microbial metabolism under PFAS stress. Existing GSMM studies have primarily focused on understanding PFAS-induced metabolic perturbations in eukaryotic systems, particularly mammalian liver metabolism [109], rather than modelling microbial degradation processes.

In principle, GSMM and constraint-based approaches can be adapted to predict microbial responses to PFAS exposure by reconstructing metabolic networks that simulate flux distributions within and between microbial species. Such models could, in future, help identify metabolic bottlenecks, cofactor limitations, and potential pathways contributing to PFAS transformation. Flux balance analysis (FBA), for example, allows quantification of metabolic flows under steady-state conditions, providing a theoretical means to evaluate how PFAS compounds might redirect carbon or energy fluxes in microbial systems [110].

At the community level, multi-species or compartmentalised metabolic reconstructions could eventually delineate interspecies dependencies, such as cofactor exchange or cross-feeding interactions that sustain degradation consortia [111]. Although these applications remain theoretical for PFAS bioremediation, they highlight how GSMM could support hypothesis generation and rational design of microbial communities once sufficient genomic and biochemical data become available [48].

Currently, GSMM should be viewed as a prospective and complementary approach rather than an established tool for PFAS biodegradation research. Its successful application will depend on the expansion of annotated microbial genomes capable of PFAS transformation, integration of experimentally validated degradation pathways, and the availability of kinetic parameters for key enzymes. These future developments may ultimately enable GSMM to bridge the gap between omics data and predictive microbial ecology for PFAS-contaminated environments.

### 4.2. Machine Learning for Predictive Modelling of PFAS-Degrading Microbial Communities

**Figure 6:**
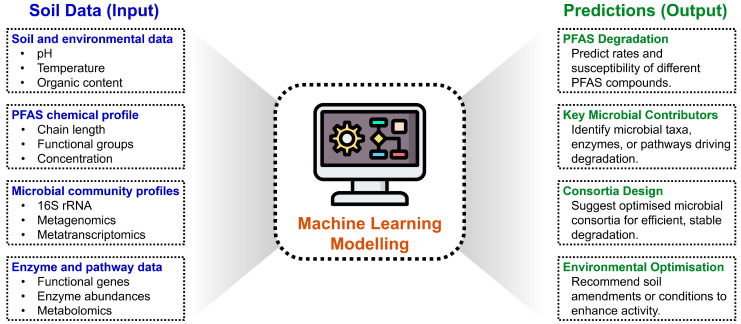
**Machine learning framework for predicting and optimising PFAS biodegradation in soil.** The model integrates a range of soil and environmental data, PFAS chemical profiles, microbial community profiles, and enzyme and pathway data as input. The framework can predict PFAS degradation rates, identify key microbial contributors, design optimised microbial consortia, and recommend environmental conditions to enhance the breakdown of these persistent chemicals.

Machine learning (ML) provides a predictive framework for understanding and designing PFAS-degrading microbial communities by integrating environmental, chemical, and biological data (Figure 6). Although still emerging, ML approaches are beginning to link PFAS fate with datasets that combine soil parameters, microbial profiles, and chemical descriptors [112].

Input datasets include soil metadata (pH, temperature, organic matter content), PFAS profiles (chain length, functional groups, concentration), and microbial community data from 16S rRNA sequencing, metagenomics, or metatranscriptomics, optionally complemented by enzyme or pathway abundance data. These heterogeneous inputs are converted into machine-readable features through one-hot encoding, embeddings for microbial taxa, and chemical fingerprints for PFAS compounds. Predictive models such as Random Forests, Gradient Boosting, Neural Networks, and Graph Neural Networks can capture complex relationships among microbes, enzymes, PFAS compounds, and environmental factors [113]. Generative models and Bayesian optimisation further support the rational design of synthetic consortia [114].

By combining mechanistic and computational approaches, predictive modelling can forecast degradation outcomes and optimise bioremediation strategies. Dynamical models that incorporate enzyme kinetics, microbial growth rates, and substrate concentrations simulate temporal dynamics of consortia, predicting interactions between primary degraders, such as strains transforming PFOA to PFHpA, and secondary degraders responsible for further defluorination [115]. In the context of PFAS control, machine learning has shown strong predictive capacity (>80%) across studies modelling PFAS behaviour in water resources, treatment performance, and energy demand during defluorination [112]. However, direct applications to microbial PFAS degradation remain limited, and most current work focuses on identifying parameters and conditions influencing biotic and abiotic transformation processes [106].

A systems-level approach combining UPLC-HRMS-based metabolomics with 16S rRNA and ITS sequencing provides detailed mapping of biochemical intermediates and their interactions with microbial communities. Integrating these experimental insights with predictive models allows the discovery of unexpected pathways, the refinement of consortia design, and the optimisation of remediation strategies, creating a robust framework applicable from laboratory-scale studies to field interventions [106,112]. Such integration of ML with experimental and omics-driven data remains a developing frontier, where future models may progressively incorporate microbiome–metabolite–PFAS relationships to support the rational design of microbial consortia with enhanced PFAS-degrading capabilities and adaptive performance under variable environmental conditions.

## 5. Prospects and Challenges

PFAS bioremediation in soils shows promise through microbial consortia enhanced with soil amendments and coupled with phytoremediation. Yet, challenges persist, including limited understanding of PFAS transformation pathways and difficulties in culturing stable degraders. Practical and regulatory barriers, such as consortium stability in heterogeneous soils, risks of gene transfer, and ecological impacts, must also be addressed. Progress will require integrated approaches combining microbiology, systems biology, and regulatory oversight to develop safe and effective strategies.

### 5.1. Soil Bioremediation Systems

Laboratory microcosms, soil amendments, and phytoremediation-coupled microbial consortia collectively highlight the potential of integrative strategies to overcome PFAS recalcitrance in soils. These approaches demonstrate that synergistic interactions between microbes, plants, and soil matrices are key to driving effective and sustainable PFAS degradation at both experimental and field scales.

#### 5.1.1. Laboratory Microcosms with Soil Microbial Consortia

Microbial consortia are increasingly recognised as powerful biocatalysts for PFAS transformation, challenging the long-held view of their complete biological recalcitrance. For example, a *Hyphomicrobium*-dominated consortium (46.7%) achieved a 56.7% reduction in PFOS within 20 days under co-metabolic conditions, illustrating how syntrophic metabolism can overcome the substantial thermodynamic barrier of C–F bond cleavage (485 kJ mol^−1^) [116]. Importantly, 53% of consortium members in this study were uncultivated, underscoring the vast untapped potential of environmental microbiomes [117]. Comparative evidence highlights that top-down functional screening from contaminated soils consistently outperforms bottom-up synthetic assembly, with enriched consortia reaching >90% degradation efficiency for PFAS precursor compounds [118]. A meta-analysis of 97 independent studies further reveals clear ecological and chemical predictors of biotransformation success: aerobic conditions, elevated PFAS concentrations (>10 mg L^−1^), and reduced degrees of fluorination all correlated significantly with higher degradation rates (*p* < 0.001) [86]. Collectively, these findings suggest that PFAS transformation in soils is primarily driven by cooperative metabolism within diverse microbial consortia rather than complete mineralisation, which remains rare and largely unconfirmed under natural conditions.

#### 5.1.2. Integration with Soil Amendments

Soil amendments provide critical leverage points for improving the efficiency of microbial consortia in PFAS-contaminated environments. Among them, biochar has emerged as both an effective sorbent and a microbial scaffold. Activated biochar (5% *w*/*w*) achieved 98–100% reduction in PFAS leaching from low-total organic carbon (TOC) soils via size-selective sorption, where pores >1.5 nm accommodated PFAS molecules of 1.02–2.20 nm [119]. However, its performance diminished in high-TOC soils (23–100% reduction), likely due to competitive sorption and pore occlusion by dissolved organic matter. Multiple studies have demonstrated that biochar immobilises PFAS in soils, reducing leaching by up to 100% depending on soil type and application rate [120,121,122]. Importantly, such immobilisation may reduce apparent PFAS concentrations without degradation, leading to false positives. Therefore, adsorption controls, mass balance, and fluoride release measurements are required to confirm biodegradation [123].

Root exudates represent another pivotal factor shaping PFAS biodegradation. Humic-like substances within exudates enhanced 6:2 FTOH defluorination 2.3-fold through two mechanisms: induction of broad-spectrum oxygenases and provision of electron donors for reductive defluorination [114]. Metabolomic profiling has identified organic acids (e.g., citrate, malate), amino acids (e.g., tryptophan, tyrosine), and phenolic compounds as major exudate constituents correlating with enhanced PFAS transformation. Interestingly, sulfur limitation amplified exudate-mediated PFAS degradation, suggesting that stress-induced metabolic reprogramming can favour activation of defluorination pathways [124].

#### 5.1.3. Coupling Microbial Consortia with Phytoremediation Strategies

The integration of microbial consortia with phytoremediation provides multifunctional systems that outperform either approach alone. Constructed wetlands combining plants, biochar substrates, and microbial consortia have demonstrated synergistic PFAS removal through partitioning–degradation coupling [125]. Biochar acts as a sorbent phase that sequesters PFAS and provides microbial habitat, requiring careful differentiation between adsorption and microbial transformation [121,123]. In such systems, plants preferentially accumulate short-chain PFAS (bioconcentration factor, BCF: 0.1–10), while rhizosphere-associated microbes transform both free and plant-accumulated compounds, thereby overcoming the chain-length dependent recalcitrance of PFAS [126]. Field-scale trials show that integrated phytomicrobial systems can achieve 70–90% reductions in total PFAS concentrations, compared to 30–50% reductions by individual approaches [127]. Beyond their role in nutrient provisioning, plant root systems generate oxic–anoxic gradients that support diverse microbial degradation pathways, while mycorrhizal networks facilitate the transport of contaminants and metabolites across soil compartments, further enhancing remediation capacity [128].

### 5.2. Scientific Challenges

Despite mounting evidence of microbial PFAS transformation, the fundamental scientific challenges remain formidable. At the molecular level, only a handful of enzyme classes have been implicated, and their precise catalytic mechanisms remain elusive. At the ecological scale, cultivation and stabilisation of PFAS-degrading consortia are constrained by both intrinsic microbial traits and extrinsic soil heterogeneity, limiting reliable translation from laboratory to field conditions.

#### 5.2.1. Limited Mechanistic Understanding of PFAS Transformation

Although empirical studies have demonstrated partial PFAS biodegradation, mechanistic understanding remains fragmentary and poorly resolved. To date, only three enzyme classes, such as dehalogenases, oxygenases, and reductases, have confirmed PFAS activity, and while key catalytic mechanisms have been elucidated, particularly for fluoroacetate dehalogenases that catalyse defluorination in bulkier and highly fluorinated substrates, including PFAS, many reaction intermediates, cofactors, and energy-coupling processes remain to be fully characterised [26]. The cleavage of C–F bonds is intrinsically unfavourable (ΔG°′ = +33 kJ mol^−1^ under physiological conditions), requiring energetic coupling to exergonic reactions that remain largely unidentified [129]. Computational models suggest possible radical-mediated mechanisms involving B12-dependent enzymes or cytochrome P450 systems, but robust experimental validation is still limited. Major gaps persist, including (i) incomplete identification of metabolic intermediates for the vast majority of PFAS structures currently circulating in the environment, (ii) uncharacterised genetic determinants of defluorination, and (iii) unknown regulatory mechanisms controlling PFAS-inducible gene expression. Without mechanistic clarity at the enzyme, pathway, and regulatory levels, the rational design and optimisation of PFAS biodegradation strategies remains out of reach [7,130].

#### 5.2.2. Difficulties in Isolating, Culturing, and Maintaining Stable PFAS-Degrading Consortia

Challenges in cultivation and stability fundamentally constrain the development of PFAS bioremediation. The well-known “great plate count anomaly” is particularly pronounced in PFAS-degrading communities, where >50% of active consortium members remain uncultivable with standard techniques [77]. Even when partial enrichment is achieved, stability is difficult to sustain diversity–stability analyses reveal an inverse correlation between species richness and temporal stability (r = −0.72), highlighting trade-offs between functional breadth and operational robustness. Laboratory-to-field transitions exacerbate this instability, with community restructuring leading to as little as 23% species overlap between liquid enrichments and corresponding soil microcosms despite identical inocula [131]. Moreover, maintaining degradation capacity typically requires continuous supplementation with co-substrates (10–100 mM methanol or acetate), which adds operational complexity and cost. Finally, the stochastic nature of community assembly in heterogeneous soils produces highly variable outcomes (coefficient of variation >40%), creating barriers to reproducible scale-up and reliable field application [132].

### 5.3. Practical and Regulatory Barriers

Scaling PFAS bioremediation from controlled laboratory conditions to complex field environments remains fraught with both practical and regulatory obstacles. At the practical level, microbial activity is highly sensitive to spatial and temporal heterogeneity in soils, where shifts in pH, redox potential, and organic matter significantly disrupt the stability and persistence of introduced consortia. At the regulatory level, the deliberate release of engineered or enriched microbes raises unresolved biosafety concerns, including the risks of horizontal gene transfer and unintended ecosystem restructuring, which remain insufficiently addressed in current environmental governance frameworks.

#### 5.3.1. Stability of Microbial Consortia in Heterogeneous Soil Environments

Soil physicochemical heterogeneity fundamentally constrains the reliable deployment of PFAS-degrading consortia. PFAS bioavailability varies by more than two orders of magnitude across soil types, governed by nonlinear interactions among organic carbon content (Koc: 10^2^–10^4^ L kg^−1^), clay mineralogy, pH (optimal range: 6.5–7.5), and ionic strength [133]. Yet multi-parameter predictive models explain only ~60% of this variance, indicating that critical determinants remain unidentified. Comparative studies reveal that fungi outperform bacteria in sustaining PFAS-degrading functions under heterogeneous conditions (R^2^ = 0.38–0.53 vs. 0.02–0.03), due to their extensive hyphal networks that bridge microscale nutrient and contaminant gradients [134,135]. Furthermore, introduced consortia are often outcompeted by indigenous microbiota, with persistence reduced by 60–80% within 30 days of soil introduction [136]. These findings underscore the necessity of site-specific optimisation strategies and highlight why universal bioremediation solutions remain elusive.

#### 5.3.2. Risks of Horizontal Gene Transfer and Unintended Ecological Impacts

The potential for horizontal gene transfer (HGT) poses significant biosafety risks that require rigorous assessment. PFAS exposure has been shown to increase conjugative transfer frequency by 2.8-fold, raising the possibility of disseminating both catabolic genes and antibiotic resistance determinants [137]. Such effects may be indirectly linked to stress responses or selection pressures induced by PFAS exposure, which can enhance plasmid mobilisation and co-transfer of resistance genes, particularly under nutrient-limited or redox-fluctuating conditions. This underscores the importance of containment strategies for ex situ systems, where microbial activity and gene transfer dynamics can be closely monitored. By-products of biodegradation may exacerbate these risks: fluoride accumulation from defluorination can reach concentrations of up to 500 mg L^−1^, potentially leading to hydrofluoric acid (HF) formation, which poses additional environmental and health risks due to its solubility and reactivity [138]. High HF or mobile fluoride concentrations disrupt soil microbial activity, inhibit key enzymes, impact plant growth, and threaten groundwater quality. Neutralisation strategies have been developed to mitigate these risks: the addition of calcium carbonate (lime) or calcium hydroxide stabilises fluoride by converting it into insoluble forms such as calcium fluoride or fluorapatite. Hydroxyapatite amendments and soil washing (e.g., using 3 M HCl) can further reduce bioavailable fluoride levels [139].

Long-term PFAS exposure also restructures microbial communities toward tolerant taxa such as *Bacteroidetes* and *Acidobacteria*, though the ecological and functional implications of this shift remain poorly understood. Current monitoring tools, primarily 16S rRNA amplicon sequencing, are insufficient to track mobile genetic elements, gene flow, or evolutionary trajectories of degradative functions. Regulatory frameworks currently lack explicit provisions for the deliberate release of engineered microbial consortia, and existing risk assessment models fail to capture long-term ecosystem dynamics or the low-probability, but high-impact events associated with rare HGT events [140].

## 6. Conclusions and Future Perspectives

PFAS contamination in soils represents a persistent and complex environmental problem due to the high chemical stability of carbon–fluorine bonds and the limited natural biodegradability of these compounds. Traditional single-strain remediation approaches are often insufficient to achieve significant PFAS removal, emphasising the critical role of microbial consortia, which utilise cooperative metabolism, metabolic division of labour, and cross-feeding of intermediates to enhance transformation efficiency. Investigations of natural consortia in PFAS-impacted soils have revealed microbial adaptation to contaminant stress, implicating oxygenases, dehalogenases, and co-metabolic pathways, while biofilm formation provides structural stability and protection in heterogeneous soil environments. Incorporating the soil–groundwater ecosystem perspective further highlights how vertical fluxes and subsurface redox gradients shape microbial activity and PFAS mobility. Strategies to enhance biodegradation include targeted enrichment and selection of keystone degraders, integration with soil amendments such as biochar and nutrient supplements, coupling with phytoremediation, and precision engineering through CRISPR and genome-scale metabolic modelling. These approaches demonstrate that system-level interventions can significantly improve defluorination rates and overall PFAS mineralisation in both laboratory and field settings.

Significant challenges remain, including incomplete mechanistic understanding of enzymatic and metabolic pathways, difficulty in isolating and maintaining stable PFAS-degrading consortia, and variability in microbial performance across heterogeneous soils. Additional uncertainty arises from PFAS vertical transport within soil profiles, where physicochemical gradients and nutrient limitations may constrain microbial activity. Practical implementation is further constrained by competition with indigenous microbiota, fluctuations in soil chemistry, and potential ecological risks associated with horizontal gene transfer or accumulation of toxic intermediates such as fluoride. Future research should prioritise cultivation-independent methods, including metagenomics, single-cell genomics, and machine learning-guided prediction of metabolic interactions to access uncultivated degraders and optimise community functionality. Integrating microbial, chemical, and plant-based strategies with advanced monitoring and predictive risk assessment frameworks will be essential for developing bioremediation systems that are both effective and ecologically safe. The successful deployment of PFAS bioremediation technologies will depend on interdisciplinary integration of microbiology, systems biology, environmental engineering, and regulatory oversight to ensure sustainability, resilience, and public safety across soil–groundwater ecosystems.

## Figures and Tables

**Table 1:** Reported studies on PFAS biodegradation by microbial consortia and single strains.

PFAS Type	Consortium/Strain	Source of Consortium/Strain	Experimental Conditions	Degradation Products	Degradation Efficiency (%)	Reference
***Single Strains***						
8:2 FTOH	*Pseudomonas* strains: *Pseudomonas butanovora* (butane oxidizer) and *Pseudomonas oleovorans* (octane oxidizer)	Environmental bacterial isolates (octane-contaminated and 1-butanol-contaminated sites)	30 °C, aerobic, 40 mg/L, 28 d	8:2 FTUCA, 7:2 ketone, 7:2 sFTOH, PFOA, PFHxA	78%	[60]
6:2 FTOH	Three alkane-degrader (*Mycobacterium vaccae* JOB5, *Pseudomonas oleovorans*, and *Pseudomonas butanovora*) and one fluoroacetate-degrader (*Pseudomonas fluorescens* DSM 8341)	Soil isolate	pH 7, aerobic, 4.125–100 mg/L, 28–90 d	5:3 FTCA, PFHxA, PFBA, 4:3 FTCA, PFPeA	88%	[61]
PFHxS	*Pseudomonas* strains PS27, PDMF10	PFAS-contaminated environmental matrices	27 °C, aerobic, 20 mg/L, 5 d	PFHxS	40%	[62]
PFOA	*Acidimicrobium* sp. A6	Acidic wetland soil isolate	pH 4.5–5, anaerobic, 25–30 °C, 18–150 d; electrochemical & enrichment setups	HFBA, PFPeA, PFHxA, PFHpA, PFBA, F^−^	50–77%	[10,11]
PFOA	*Pseudomonas parafulva* YAB1	Soil near a perfluorinated compound production plant	pH 7, 30 °C, inorganic salts + 1 g/L glucose, aerobic, 4 d	F^−^	48%	[63]
6:2 FTOH	*Gloeophyllum trabeum*	White-rot fungi	30 °C, aerobic, 3 mg/L, 28 d	PFCAs and 6:2 FTOH metabolites	12% and 51%	[64]
PFOS	*Pseudomonas plecoglossicida* 2,4-D	waste from petrochemical production	pH 7, 28 °C, mineral medium, 6 d	PFOS	75%	[65]
6:2 FTSA	*Rhodococcus jostii* RHA1	Lindane-contaminated soil in Japan	pH 7, 30 °C, sulfur-free mineral salt medium, aerobic, 7 d	6:2 FTUSA, 6:2 FTOH and 6:2 FTSA	99%	[66]
6:2 FTOH, PFOA	*Cunninghamella elegans*	Soil fungi used for xenobiotic degradation	28–30 °C, 150 rpm, 72 h incubation; 0.1 mg/L of 6:2 FTOH, PFOA	5:3 FTCA is the major product, with another 10 more short-chain products	100% biotransformation of 6:2 FTOH; 90% biotransformation of PFOA	[36,50,55]
***Microbial Consortia***						
PFOS	Bacterial consortium: *Paracoccus* (72%), *Hyphomicrobium* (24%), and *Micromonosporaceae* (4%)	Activated sludge	Encapsulated activated sludge consortium; initial PFAS 2 mg/L.	PFOS, PFBS, 3,3,3-trifluoropropionic acid	52–74%	[13]
PFOS	Microbial consortium (Archaea and Bacteria Domains)	Domestic sewage	PFOS (100 μg L^−1^) incubated for 10 days at 35 °C and pH 6	PFOS: C4F9CHO (2,2,3,3,4,4,5,5,5-nonafluoropentanal)	24%	[67]
6:2 FTNO, 6:2 FTSA, 6:2 FTAA	Aerobic sludge microcosms	Activated sludge	25 °C, 100 d, 10 μg/L	Multiple products from FTSA (7 products) and FTNO (15 products)	100%	[68]
PFOA	Microbial electrosynthesis system (MES)	Mixed microbial biofilm	35 °C, 5 d, 1.5–10 ppm	PFOA: C_6_HF_13_, C_3_F_3_H_4_COOH, C_6_F_3_COOH, C_6_F_9_H_4_COOH	91% within 120 h	[69]
PFOS, PFBS, TFA, 6:2 FTSA	Anaerobic microbial consortium	Mixed anaerobic wastewater sludge	up to 500 mg L^−1^ PFOS; 110 weeks of incubation	Anaerobic (≤500 mg/L PFAS, FTSA; 3.4-year incubation for PFOS) or aerobic (32-week incubation for short-chain PFASs: PFBS and TFA).	No detectable microbial degradation	[70]
PFOA, PFOS	*Chlorella vulgaris* and *Scenedesmus obliquus*	Algal consortium	pH 7, 25 °C, 16:8 h light/dark, 90 μmol photons/m^2^s; 10 mg/L; 7 d	PFOA, PFOS	11–16%	[71]
AFFFs, 6:2 FTSA, and 6:2 FTAB	*Gordonia* sp. strain NB4-1Y	Aerobic soil	7-day, 34.2 mg/L	FTAB, FTSA, and AFFFs	99.9% of 60 μM 6:2 FTSA, 70.4% of 60 μM 6:2 FTAB	[72]
PFOA	Aerobic microbial community	*Stenotrophomonas*. *Bacillus*, *Pseudomonas*, and *Brevundimonas*	500 µg/L, 96 h	PFOA, TOC,	79.7 ± 9.4%, 57.4 ± 3.4%, and 57.6 ± 12.9%	[73]
PFASs, PFBA	Mixed algal solution (Chlorophyta + Bacillariophyta)	Bacteria-algae symbiotic aquatic ecosystem	PFAS 2–200 μg/L, 90 d	PFBA, PFTeDA	PFPeA (13.21–13.99%), (10.04–10.50%), PFBA (10.38–14.68%), and PFTeDA (10.33–15.96%)	[74]
PFOA, PFOS	*Chlorella* sp. (algal consortium)	Algal culture	23 °C, 12:12 h light/dark (5000 l×); 320 mg/L PFOA, 160 mg/L PFOS; 7 d	PFOA and PFOS uptake	PFOA uptake: 0.89–0.66%, PFOS uptake: 1.21–0.95%	[75]
PFAAs, PFBA, PFBS, PFOA	*Ulothrix* + *Potamogeton crispus* (phytoremediation)	Submerged aquatic plants	PFAS 21.5 mg/L	Higher BAF of PFBA and PFBS, while lower BAF of PFOA and PFOS	40% and 81%	[76]

PFOA, perfluorooctanoic acid; PFOS, perfluorooctane sulfonic acid; 6:2 FTSA, 6:2 fluorotelomer sulfonic acid; 6:2 FTOH, 6:2 Fluorotelomer alcohol; 6:2 FTAB, 6:2 fluorotelomer sulfonamidoalkyl betaine; MES, microbial electrosynthesis system; 6:2 FTSA, 6:2 Fluorotelomer Sulfonic Acid; 6:2 FTAA, 6:2 Fluorotelomer Acetic Acid; FTNO, 6:2 FTNO, 6:2 Fluorotelomer Nitro Alcohol; PFBA, Perfluorobutanoic Acid; FTUCA, Fluorotelomer Unsaturated Carboxylic Acid; PFpeA, Perfluoropentanoic acid; PFHeA Perfluoroheptanoic acid; TFA, trifluoroacetic acid; F^−^, fluoride ion.

## Data Availability

All relevant data are included in the paper.
